# Effect Evaluation of *Sahtak bi Sahnak*, a Lebanese Secondary School-Based Nutrition Intervention: A Cluster Randomised Trial

**DOI:** 10.3389/fnut.2022.824020

**Published:** 2022-03-16

**Authors:** Liliane Said, Jessica S. Gubbels, Stef P. J. Kremers

**Affiliations:** ^1^Department of Health Promotion, NUTRIM School of Nutrition and Translational Research in Metabolism, Faculty of Health, Medicine, and Life Sciences, Maastricht University, Maastricht, Netherlands; ^2^Department of Nutrition and Food Sciences, Faculty of Arts and Sciences, Lebanese International University, Bekaa, Lebanon

**Keywords:** Intervention Mapping, obesity, adolescents, nutrition education, dietary knowledge, dietary adherence, cluster randomised controlled trial

## Abstract

**Objective:**

To evaluate the effectiveness of *Sahtak bi Sahnak* on dietary knowledge and adherence to dietary guidelines in Lebanese adolescents.

**Design/setting:**

A cluster randomised controlled trial was conducted in public and private secondary schools located in urban and rural regions in Lebanon.

**Participants:**

Sixteen secondary schools including 1,572 adolescents were randomly assigned to the intervention (*n* = 739) or control group (*n* = 833).

**Intervention:**

*Sahtak bi Sahnak* is an educational school-based intervention dedicated to improving dietary adherence to nutritional guidelines, increasing the level of dietary knowledge, and preventing the development of obesity during adolescence. It was systematically designed based on the Intervention Mapping framework. The total length of the intervention was around seven educational sessions, until all of the 11 lessons were covered. Each education session lasted 20–40 min.

**Main Outcome Measures:**

Dietary knowledge and adherence levels were measured at baseline and post-intervention using validated questionnaires.

**Statistical Analysis:**

Multivariate multilevel regression models were used to examine intervention effects on outcomes, controlled for background characteristics (i.e., age, gender, location, type of school, grade, BMI *z*-score).

**Results:**

The intervention group showed significant improvements in total dietary knowledge (*B* = 12.74, *p* < 0.001) and intake of healthy items (*B* = 1.89, *p* < 0.001), compared to the control group. Intake of unhealthy items decreased significantly (*B* = −1.43, *p* < 0.001), compared to the control group. These results were adjusted for age, gender, type of school, location, BMI *z*-score, study group, and score at baseline.

**Conclusion and Implications:**

*Sahtak bi Sahnak* is an effective and culturally appropriate school-based intervention for targeting diet among a variety of Lebanese adolescents.

## Introduction

According to the World Health Organization (1), paediatric obesity is one of the most serious global health problems of the current century. In the last 40 years, the number of obese children and adolescents increased by more than 11 times, reaching a total of 124 million in 2016 (2). The Arab countries are no exception as the paediatric obesity prevalence reached 22.8% in Kuwait and 7.5% in Tunisia among 10–19-year-old adolescents in 2016 (3). In Lebanon, the prevalence of obese adolescents of the same age category increased from 7.9% in 2000 to 12.2% in 2016 (3). Childhood obesity can lead to several physical (e.g., insulin resistance, cardiovascular diseases, etc.) and mental complications (e.g., low self-esteem, depression, etc.), sometimes lasting into adulthood (4, 5). It is often caused by an imbalance between energy intake and energy expenditure (4). Still, efforts to resolve this epidemic have not yet been able to reverse the growth of this problem (6).

Health experts have elaborated several comprehensive recommendations and strategic objectives to plan and implement effective interventions addressing paediatric obesity. This includes specifying the intervention setting and determinants to be changed. Researchers confirm that obesity prevention efforts should target children and that such interventions should be based on appropriate theoretical frameworks (7, 8). In addition, the Commission on Ending Childhood Obesity recommends implementing programmes to promote healthy eating habits and reducing the intake of unhealthy foods and sugar-sweetened beverages by children and adolescents (9). As for the intervention setting, schools play an important role in nutrition education as they can improve healthy eating habits for lifetime prevention of obesity (9). In addition, integrating nutrition education as part of the school’s curriculum and improving nutrition literacy is in line with schools’ pedagogical mission (10). Several studies showed that school-based nutrition interventions improved dietary knowledge (11, 12), eating patterns (11, 13) and reduced overweight and obesity (13, 14). Cluster randomised trials (CRT) are widely used to evaluate health promotion interventions (15). They present several advantages as they are applicable in school settings and tend to reduce the exchange of information between the research groups (intervention and control group) (16).

When designing an effective obesity prevention intervention in a school setting, it is important to consider an appropriate theoretical framework, relevant and modifiable determinants, and appropriate application methods. The Intervention Mapping (IM) (17) provides a logical and evidence-based process to develop, implement, and evaluate a health promotion intervention. It also allows the health promotors to select the appropriate theoretically informed method to influence the causal determinants that regulate the eating habits. The literature identified a broad range of determinants influencing eating habits (e.g., individual factors, parental influence, etc.) (18). The needs assessment in IM revealed that the most relevant and modifiable determinants related to eating habits among adolescents are dietary knowledge, self-efficacy, attitude, and skills (19). Dietary knowledge (also known as nutrition knowledge) is an important and modifiable determinant of eating habits (18). It may also serve as a pre-requisite for other mentioned determinants (e.g., skills). Several studies have shown that higher levels of nutrition knowledge are significantly and positively correlated with healthier eating habits (20–22). In Lebanon, a previous study reported that Lebanese adolescents had relatively low levels of dietary knowledge and adherence to dietary recommendations (23, 24). Despite the alarming obesity rates and the low dietary knowledge and adherence levels among Lebanese adolescents, very few interventions have aimed to prevent paediatric obesity in Lebanon, focusing solely on children aged 9–11 years using a 3-month school-based programme (12, 25). To our knowledge, no nutrition interventions have yet been implemented in Lebanese high schools, and none focused on both urban and rural regions. Therefore, we developed *Sahtak bi Sahnak* (صحتك بصحنك; in English: “Your health on your plate”), the first educational school-based nutrition intervention targeting Lebanese adolescents aged 15–18 years, living in urban and rural regions, to prevent obesity (19). This is also the first theory- and evidence-based obesity-prevention intervention following the IM framework in the Arab world. The purpose of the current paper is to evaluate the effectiveness of *Sahtak bi Sahnak* in improving both dietary knowledge and dietary adherence levels in a cluster randomised controlled trial. The study objectives pertain to the individual participant level.

## Materials and Methods

### Study Design and Participants

The current cluster randomised controlled trial was conducted between October 2017 and March 2018 and targeted 15- to 18-year-old Lebanese adolescents attending public and private high schools in Beirut (urban region), Baalbeck and Rayak (both rural regions) in Lebanon. Beirut represents the urban region as it is the capital and largest city of Lebanon. Baalbeck and Rayak represent the rural region, located in the Bekaa region, which is considered Lebanon’s most important agricultural region. Schools, representing the cluster unit, were randomly selected from the 96 high schools located in the selected regions with standard day-time schedules (classes finishing by 15:00 in the afternoon at the latest). During the recruitment phase, the schools were divided according to the indicated study location (rural vs. urban) and type of school (public vs. private). All the public secondary schools located in the rural region (i.e., Baalbeck and Rayak; *n* = 4), agreed to participate. In parallel, private schools from the rural region were randomly selected and approached until four of them accepted to participate. Similarly in the urban region (i.e., Beirut), public schools were randomly selected and approached. The recruitment of public schools in Beirut was stopped when the required sample size of six schools was met. As for the urban private schools, two private schools accepted to participate.

**FIGURE 1:**
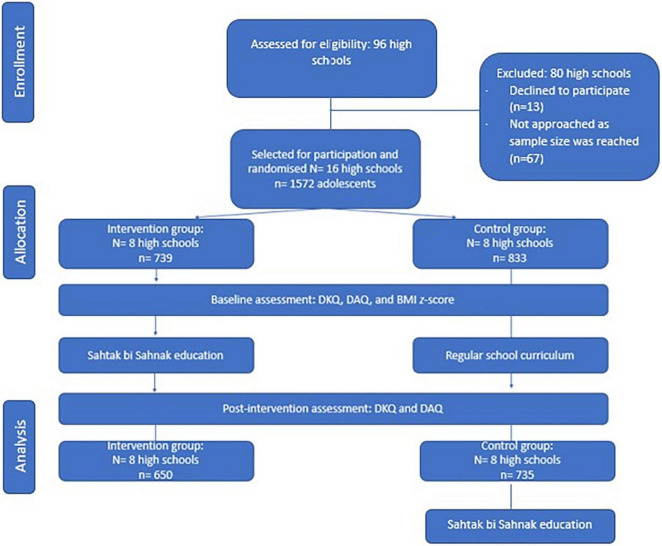
Flow chart of the study design. DKQ, Dietary Knowledge Questionnaire; DAQ: Dietary Adherence Questionnaire; BMI: body mass index.

Based on the pre-determined required sample size, a total of 16 schools were recruited. The required sample size (*n*) was calculated based on an expected mean change in nutrition knowledge (one of the main outcomes) based on previous literature of 18.2% with a standard deviation of 0.54 (26), a power level of 80%, and a significance level of 5%. This resulted in *n* = 139 per group (27), equalling *n* = 278 for both groups. However, it is important to note that schools differ in the numbers of students per school and per class. Thus, to obtain a diverse sample with participants from both regions (urban and rural), from both common types of schools in Lebanon (private and public) and from both selected grades (grades 10 and 11) in both study groups, we recruited more participants and ended up with a total of 1,572 adolescents from 16 schools (see Figure 1). The type of school is also interpreted as an indicator for the socio-economic status of the participants, as private schools have higher tuition fees compared to public schools.

The 16 schools (representing 50% of public high schools and 8% of private high schools) that accepted the invitation to participate were randomly divided into two groups (intervention and control), with the restriction of balancing public and private high schools as the number of public schools was higher compared to private ones (see Figure 1). During the recruitment phase, the lists of public and private secondary schools located in Beirut city, Baalbeck, and Rayak were obtained from the Lebanese Ministry of Education and Higher Education (MEHE). The principal investigator (PI) randomly selected the schools from the list and visited them to meet the school principal in person. After receiving the consent of the school principal [i.e., by signing the consent letter after reading the information letter (19)], all students present at the moment of data collection were approached. Participation was voluntary. No incentive nor compensation was offered to schools or participants. None of the students refused to participate. However, the number of participants differed at different data collection moments due to student absenteeism. Inclusion criteria for the participating adolescents included: (1) having a Lebanese nationality and being enrolled in a Lebanese public or private high school located in Beirut, Baalbeck, or Rayak; (2) aged 15–18 years; (3) having full cognitive, psychiatric and physical ability to communicate (as reported by parents or by school administration); (4) not having any chronic or genetic diseases (as reported by parents or by school administration).

To avoid bias, participants were not pre-informed about any data collection moment. During the first meeting with adolescents, the PI explained the research goals and the procedure related to data collection. Students’ consent was also orally confirmed by the PI before administering the questionnaire. The questionnaire further clearly stated that once it was filled in and handed in, this meant that the adolescent accepted and assented to participate in the study. The study was conducted in accordance with the Declaration of Helsinki, and the protocol was approved by the Lebanese MEHE (15465/3/2016; date: 06/10/2017) and the Institutional Review Board of the Lebanese International University (LIUIRB-171212-LS1).

Several measures were considered to decrease the risk of exposing the control group to the intervention and subsequent bias. Randomisation into the intervention and control group was conducted by the PI (first author) at the school level, so that participants from the same school would be exposed to the same information (28), following stratified randomisation procedures (toss of a coin) with a 1:1 allocation. The intervention schools received the intervention, while the control schools provided their education according to the regular curriculum.

### Data Collection

#### Instruments

##### Background Characteristics

The socio-demographic information was assessed at baseline and included the following: date of birth to calculate the age in years; gender (boys/girls); school class (grade 10/grade 11); school’s name to determine type of school (public or private); and school address to determine region (urban or rural).

##### Anthropometric Measurements

At baseline, adolescents were weighed with light clothes and no shoes on, to the nearest 0.1 kg, using a digital weight scale (EKS). Height was measured without shoes and recorded to the nearest 0.1 cm, using a portable stadiometer (Numed). Weight and height were measured using standardised protocols and procedures (29) and calibrated equipment. In the current study, the gender- and age-specific BMI *z*-scores were calculated using the WHO AnthroPlus software (30). The BMI is a relatively inexpensive, easy-to-obtain, non-invasive and quick method for detecting obesity (31, 32). However, as it is not an accurate tool for monitoring change in adiposity in children and adolescents (33), and due to the relatively short duration of the intervention, the BMI was measured at baseline only, and not taken into account as an outcome.

##### Dietary Knowledge

A validated Dietary Knowledge Questionnaire (DKQ) was administrated to adolescents at baseline (T_0_) and post-intervention (T_1_). The DKQ includes 23 questions divided to five parts (34): (1) knowledge of dietary recommendations; (2) knowledge of nutrient sources; (3) knowledge of common misconceptions in nutrition; (4) using knowledge of nutrition to make dietary choices; and (5) knowledge of associations between nutrition and diseases. Each question has three to five answer options and only one correct answer. The maximum possible score is 56, reflecting an extremely high level of dietary knowledge, and the minimum score is zero. The questionnaire showed an acceptable internal reliability (35), as Cronbach’s alpha was 0.82 (23).

##### Dietary Adherence

A validated self-reported Dietary Adherence Questionnaire (DAQ) (34) was administrated to adolescents at baseline (T_0_) and post-intervention (T_1_), to assess their level of adherence to the dietary recommendations from the American Heart Association (36) and the Dietary Reference Intakes (37), as Lebanese equivalents are lacking. The DAQ included 30 questions. Two scores were calculated: a healthy items score (ranging from 0 to 37) and an unhealthy items score (ranging from 1 to 38). The internal reliability was acceptable, as Cronbach’s alpha was 0.64 for healthy items and 0.61 for unhealthy items (23). Examples of healthy items include healthy food choices (e.g., wholemeal bread, vegetables, and fruits) and healthy meal patterns (e.g., breakfast consumption and eating three meals). Unhealthy items include unhealthy food choices (e.g., soft drinks, sweets, and fried meat) and unhealthy meal patterns (e.g., eating outside of the home).

#### Data Collection Procedures

The school-based data collection was performed by a dietitian (PI) and a trained research assistant. The questionnaires, administered in Arabic (native language of the participants), were specifically designed to suit the Lebanese adolescent population, having been pre-tested and validated using qualitative (i.e., cognitive interview) and quantitative methods among Lebanese adolescents (34). In addition, quality control measures regarding the training, data collection and data entry monitoring (e.g., data curation, double entry, and range checks for data values) were applied and complied with the CONSORT guidelines (see Supplementary Material). The collected data, at baseline (T0) and after 45–90 days of follow-up (T1), are described in more detail below. The follow-up moment corresponded to one week after completing the intervention.

### Intervention

The *Sahtak bi Sahnak* intervention (صحتك بصحنك) was a school-based intervention targeting Lebanese adolescents. *Sahtak bi Sahnak* was developed using the IM framework (17), enabling the development and evaluation of theory and evidence-based health promotion programmes. Its main aim was to prevent the development of obesity during adolescence. The sub aims were: (1) to improve dietary adherence to nutritional guidelines (36, 37); and (2) to increase the level of dietary knowledge. The implementer of the intervention was a dietitian who delivered the intervention directly to adolescents in classroom settings at the participating schools. The educational material was specifically designed to suit our target population. It was written in Arabic language, which is the native language of the participants, and it took into consideration their cultural and traditional values and dietary patterns. The intervention lessons covered various topics including benefits of healthy eating, principles of healthy eating, lipids, physical activity, healthy weight, challenges of healthy eating and physical activity practice, vitamins and minerals, importance of water, nutrition facts label, diets, and food safety. The development and design of the intervention are described elsewhere in more detail (19).

Once the school was enrolled in the programme, each school principal designated one or two sessions per week, depending on the school’s courses, exam schedule, and holidays. The total length of the intervention was around seven educational sessions, until all the 11 lessons were covered, spread over an average of 2 months. Each education session lasted 20–40 min. Each lesson tackled a different topic about nutrition. An interactive discussion followed the lesson explanation to answer any additional questions and to recapitulate what was learnt. The current intervention targeted several types of knowledge, such as declarative knowledge (i.e., awareness) and procedural knowledge (i.e., learning how to make the right choices) (17). In addition, attitude, skills (e.g., learning to plan a healthy lunch menu), and self-efficacy were addressed, as well. The education sessions focused on a healthy lifestyle rather than explicitly on obesity, to target participants with different BMI *z*-scores, as 10.7% of the target group are underweight (23) and would also benefit from a healthy lifestyle. In addition, this helped in avoiding weight stigma among teenagers. Hence, the main focus of this intervention was to promote healthy eating patterns. In addition to the classroom lessons, reader-friendly, attractive, and informative booklets were distributed to each participant to reinforce the learning process. At the end of the study and for ethical reasons, the control group received the nutrition education after completing the data collection at T_1_ (post-intervention).

### Statistical Analyses

Data were entered and analysed using the Statistical Package for Social Sciences (IBM SPSS Statistics for Windows, Version 25.0. Armonk, NY: IBM Corp.). Descriptive statistics were used to analyse the participants’ characteristics and outcomes. There were three dependent variables: (1) healthy items score, post-intervention; (2) unhealthy items score, post-intervention; and (3) total knowledge score, post-intervention. The independent variables included the following: (1) age; (2) gender; (3) grade (grade 10/11); (4) type of school (public/private); (5) location (urban/rural); (6) BMI *z*-score; (7) study group (intervention/control); and (8) score at baseline. Chi-square tests were applied to analyse the variation in frequency of the categorical background characteristics (e.g., gender) between the intervention and control group. Independent *t*-tests were used to compare differences between groups (intervention vs. control) at baseline for continuous variables (e.g., BMI *z*-scores). The intervention effect was analysed using a multilevel analysis with random intercepts, adjusting for significant covariates found in the baseline analysis as well as baseline scores for the outcome variables, with total dietary knowledge and healthy items scores as dependent variables. As these analyses were adjusted for baseline scores, the reported regression coefficients reflect difference between intervention and control group in the change between baseline and follow-up. Three levels were included: (i) level 1: adolescents; (ii) level 2: schools; and (iii) level 3: location (Beirut/Baalbeck/Rayak). Where the random intercept was non-significant in the multilevel analysis, a multivariate regression model was performed to test the intervention effect, adjusting for the same significant covariates. In addition, a multilevel analysis was performed to evaluate the interaction between background variables and the intervention variable associated with the dependent variables (i.e., post-intervention scores of dietary knowledge, healthy items, and unhealthy items). Separate multivariate regression analyses were conducted to evaluate the intervention effect in different subgroups (based on gender, location, type of school, grade, weight status, and score at baseline), for which continuous variables were recoded into categories as follows: BMI *z*-score was classified into: <−3 indicating severe malnutrition; −2 to −2.9 indicating moderate malnutrition; −1 to −1.99 indicating mild malnutrition (38); −0.99 to 1.03 indicating normal weight; 1.04 to 1.63 indicating overweight; 1.64 to 2.32 indicating obesity; and ≥2.33 indicating severe obesity (39). Knowledge score was recoded into: 1 (= low), range 0–28; and 2 (= acceptable), range 29–56. All models were adjusted for age, gender, class/grade, type of school (public vs. private), location (urban vs. rural), BMI *z*-score, and score at baseline. The regression method used was forced entry, and missing values were excluded listwise. Prior to conducting the regression analysis, the data was checked to meet the following assumptions: (1) the relationship between the independent and dependant variables was linear; (2) there was no multicollinearity in the data; (3) the values of the residuals were independent; (4) the variance of the residuals was constant; (5) the values of the residuals were normally distributed; and (6) there were no influential cases biassing the model. *p*-Values below 0.05 were considered statistically significant.

## Results

The school recruitment phase lasted from January till September 2017, while the date collection started in October 2017 and ended in March 2018. The background characteristics of the 1,572 participating adolescents are shown in Table 1. Overall, there were more girls (66.1%) than boys, more participants from the rural regions (68.1%) compared to urban regions, and more students from public high schools (71.2%) than from private schools. A total of 16 secondary schools participated in the study of which 8 schools were located in Beirut (6 public and 2 private), 6 schools were located in Baalbeck (3 public and 3 private), and 2 schools in Rayak (1 public and 1 private).

**TABLE 1:** Background characteristics of the participants.

	Intervention	Control	Total
	(*N* = 739)	(*N* = 833)	(*N* = 1,572)
Age, mean (SD)	15.8 (0.8)	15.8 (0.8)	15.8 (0.8)
Gender***, *n* (%)			
Boys	390 (52.8)	143 (17.2)	533 (33.9)
Girls	349 (47.2)	690 (82.8)	1039 (66.1)
Location***, *n* (%)			
Beirut (U)	327 (44.2)	174 (20.9)	501 (31.9)
Baalbeck (R)	346 (46.8)	517 (62.1)	863 (54.9)
Rayak (R)	66 (8.9)	142 (17.0)	208 (13.2)
Type of school***, *n* (%)			
Public	390 (52.8)	729 (87.5)	1119 (71.2)
Private	349 (47.2)	104 (12.5)	453 (28.8)
Grade***, *n* (%)			
Grade 10	383 (52.3)	514 (61.9)	897 (57.4)
Grade 11	349 (47.7)	316 (38.1)	665 (42.6)
BMI *z*-score**, mean (SD)	0.5 (1.2)	0.4 (1.1)	0.4 (1.2)

*Percentages are calculated according to the number of participants with non-missing values for each item. Chi-square tests and independent t-tests were performed to detect statistical differences between groups at baseline.*

*U, urban; R, rural; SD, standard deviation.*

**p < 0.01; ***p < 0.001.

When comparing the intervention and control group at baseline, there were significant differences in terms of gender (*p* < 0.001), location (*p* < 0.001), type of school (*p* < 0.001), and grade (*p* < 0.001). There was a significant difference in the average BMI *z*-score between the intervention (*M* = 0.5, SD = 1.2) and the control group (*M* = 0.4, SD = 1.1); *t*(1,338) = −2.72, *p* = 0.007. Additionally, there was a significant difference in the baseline total knowledge score between the intervention (*M* = 26.18, SD = 8.10) and the control (*M* = 27.56, SD = 6.8) groups; *t*(1,417) = 3.60, *p* < 0.001. There was also a significant difference in the baseline healthy items score between the intervention (*M* = 8.38, SD = 3.76) and the control group (*M* = 9.22, SD = 3.48); *t*(1,440) = −4.42, *p* < 0.001. Furthermore, the average unhealthy items score at baseline of the intervention group (*M* = 6.71, SD = 3.23) was significantly higher compared to the control group (*M* = 6.08, SD = 3.12); *t*(1,436) = 3.72; *p* < 0.001 (Tables 1, 2). Therefore, these variables were adjusted for in the multiple regression and multilevel analyses.

**TABLE 2:** Means and standard deviations (SD) of the total dietary knowledge score, healthy items score, and unhealthy items score and the intervention effect on the three scores.

	Intervention Unadjusted mean (SD)		Control Unadjusted mean (SD)		*B*	95% CI
	T_0_	T_1_	T_0_	T_1_		
Dietary knowledge score	26.18 (8.10)^a^	38.90 (7.88)	27.56 (6.8)^a^	26.51 (7.83)	12.74***	10.36–15.12
Healthy items adherence score	8.38 (3.76)^b^	10.02 (2.97)	9.22 (3.48)^b^	8.31 (3.11)	1.89***	0.97–2.81
Unhealthy items adherence score	6.71 (3.23)^c^	4.85 (2.34)	6.08 (3.12)^c^	5.74 (2.53)	−1.43***	−1.75 to −1.11

Score ranges: dietary knowledge score: 0–56, healthy adherence score: 0–37, and unhealthy adherence score: 1–38. Multilevel analyses, with random intercepts and including three levels (level 1: adolescents; level 2: schools; level 3: location), were used to analyse the intervention effect on the total dietary knowledge and healthy items scores. Multivariate regression model was performed to test the intervention effect on the unhealthy items score. All models were adjusted for gender, class, type of school (public vs. private), location (urban vs. rural), BMI z-score, and score at baseline. ^a,b,c^Values with the same superscripts are significantly different by study group (intervention vs. control) at baseline and p < 0.001; ***p < 0.001 between the intervention and control group at T_1_.

*SD, Standard deviation; CI, confidence interval; T_0_,: baseline; T_1_, post-intervention.*

Based on the adjusted multilevel models, the intervention group showed significant improvements in total dietary knowledge (*B* = 12.74, *p* < 0.001) and in the healthy adherence score specifically (*B* = 1.89, *p* < 0.001) compared to the control group (Table 2). The total dietary knowledge score of the intervention group increased by 48.6% and the healthy adherence score increased by 19.6%, while they both decreased in the control group. The unhealthy adherence score significantly decreased (*B* = −1.43, *p* < 0.001) in the intervention group compared to the control group. The unhealthy adherence score decreased by 27.7% in the intervention group. Changes in separate DAQ items are shown in Supplementary Material.

In addition, when analysing the total dietary knowledge score, significant interactions were found between the intervention and the following background variables: grade (*p* = 0.019), knowledge score at baseline (*p* < 0.001), and weight status (*p* = 0.045). Subgroup analyses based on significant interaction between the intervention and background variables (see Table 3) showed that the intervention led to larger improvements in knowledge among the oldest children, underweight children, and children with a low baseline score. As regards healthy items adherence, a significant interaction was found between the intervention and location (*p* = 0.011). The intervention showed larger improvements among adolescents from rural regions, compared to urban regions. There was no significant differential intervention effect among public and private schools. Table 3 shows the intervention effects among subgroups having a significant interaction with the intervention (see Supplementary Table 1 for the full list of intervention effects in subgroups).

**TABLE 3:** Intervention effects on the total knowledge score and healthy items adherence score of the indicated subgroups.

	*B*	CI 95%
**Total dietary knowledge score**		
**Grade^a^**		
Grade 10	11.13***	9.89–12.36
Grade 11	13.65***	12.52–14.78
**Weight status^a^**		
Underweight	15.36***	12.82–17.89
Healthy weight	12.52***	11.41–13.62
Overweight/obese	10.61***	9.03–12.19
**Knowledge score at baseline^b^**		
Low	13.48***	12.26–14.70
Acceptable	9.87***	8.42–11.32
**Healthy items adherence score**		
**Location ^a^**		
Urban	3.26***	2.68–3.85
Rural	0.77**	0.26–1.29

Multivariate regression model was performed to test the intervention effect on the total knowledge score, healthy items score, and unhealthy items score in the indicated subgroups. All models were adjusted for age, gender, class, type of school (public vs. private), location (urban vs. rural), BMI z-score, and score at baseline. BMI classification: adolescents with a BMI z-score ≤−1 were classified as underweight; adolescents with −0.99 ≤ BMI z-score ≤ 1.03 were classified as having a healthy weight; and adolescents with a BMI z-score ≥ 1.04 were considered overweight/obese. Adolescents with an acceptable baseline knowledge score correctly answered >50% of questions, and adolescents with a low baseline knowledge score had <50% of correct answers. The interaction between background variables and the intervention variable was found to be significantly associated with the dependent variables (i.e., knowledge score and healthy items score) with ^a^p < 0.05 and ^b^p < 0.001; CI, confidence interval; **p < 0.01; ***p < 0.001.

## Discussion

The present study evaluated the effectiveness of *Sahtak bi Sahnak* in improving both dietary knowledge and self-reported adherence levels in a cluster randomised controlled trial among Lebanese adolescents in both urban and rural regions. The results showed significant improvements in both dietary knowledge and dietary adherence, across various subgroups.

Our intervention significantly and substantially increased dietary knowledge by 48.6% in the intervention group. Our effects are larger compared to another Lebanese intervention for younger children (9–11 years) (12, 25). Another educational intervention conducted in India showed that nutrition knowledge improved significantly in participants aged 15–18 years enrolled in public schools, but not in private ones (40). This could be due to the higher baseline knowledge scores of the adolescents in private schools in their sample, creating a ceiling effect in private schools (40). The current study did not show differential intervention effects among public and private schools. However, no significant baseline differences were found between the knowledge scores from private and public schools in the current sample (23), potentially explaining the lack of differential effects in the current study as there were no ceiling effects. Alternatively, the current intervention might have been more suitable for adolescents from private schools in addition to public schools, compared to the Indian intervention.

Our results also suggest that, although the intervention was effective for both grades, it was more effective in increasing dietary knowledge among grade 11 adolescents compared to grade 10 adolescents. This could be due to the generally smaller number of students per class in grade 11, enabling better management of the students (i.e., less noise) and improving the delivery of the intervention. In addition, *Sahtak bi Sahnak* was more effective in impacting the dietary knowledge of participants with a lower baseline score. This is a positive outcome because during the intervention design, specific attention was paid to simplify the nutrition information and key terms as much as possible to make it suitable for participants of all dietary knowledge levels. The intervention appears to have succeeded in this, having significant effects on adolescents with low as well as with acceptable knowledge level. Nonetheless, adolescents with an acceptable baseline knowledge level were also able to significantly benefit from the intervention. Furthermore, and surprisingly, the intervention seems to present a larger improvement among underweight adolescents compared to overweight/obese ones, even though it was initially intended to prevent paediatric obesity. This could be related to the baseline knowledge scores, as a higher BMI *z*-score was significantly positively correlated with higher knowledge score (23). The higher baseline dietary knowledge score among overweight and obese children may be attributed to a higher interest in nutrition compared to other children (21).

In addition to improving knowledge, the current intervention succeeded in significantly improving self-reported dietary adherence by increasing the healthy items score and decreasing the unhealthy items score. This is partially in line with an intervention for Tunisian adolescents (41), which showed an improvement related to the consumption of the recommended amount of fruits and vegetables. Another nutrition intervention for adolescents based on the IM framework, conducted in Ecuador, significantly reduced the intake of added sugars and unhealthy snacks. However, the breakfast and fat intake did not change (13). Our results additionally suggest that the intervention was more effective in improving the healthy items score among urban adolescents compared to rural participants, which may be caused by a reduced food availability in some rural regions (42). This could be also due to the significant difference of the healthy items score at baseline between both groups, in addition to the higher number of adolescents per class in rural schools (23).

The current study has several strengths. First, the sample size is large and diverse, covering both urban and rural areas with different socioeconomic backgrounds. Second, this is the first study in the region to evaluate the effect of an intervention that was systematically developed based on IM (17), aiming to prevent obesity. This elaborate framework allows researchers to develop interventions based on the latest findings related to theories and empirical findings, instead of randomly selecting a behaviour change approach (43). Third, data collection was performed according to standardised procedures and included valid and pre-tested questionnaires specifically designed for Lebanese adolescents (34). Fourth, the effect of *Sahtak bi Sahnak* was demonstrated using advanced and rigorous statistical tests to evaluate its effectiveness, adjusting for relevant background characteristics and the multilevel structure of the data. Fifth, the applied randomisation guarantees the internal validity for statistical tests of significance that are used to compare the intervention effects (44). Sixth, unlike other programmes (13), the current intervention can be adopted in Lebanese public and private secondary schools located in both urban and rural regions.

Some limitations should be also considered. First, the sample did not include participants from all the Lebanese regions, and thus generalizability to the rest of Lebanon might be limited, despite the significant effects in the various subgroups examined. In addition, sampling was not stratified at the individual level, e.g., for gender. However, the study did include both rural and urban regions, and both public and private schools. Second, significant differences were found between the intervention and control groups at baseline (e.g., gender, classes, location, etc.). These differences were, however, adjusted for in the statistical analyses. The analyses were not, however, adjust for class size, although class size is related to grade in Lebanon, and the analyses were adjusted for grade. Third, the dietary knowledge and adherence variables were based on self-reports rather than on objective assessment, potentially resulting in social desirability bias. However, direct objective measurements are not feasible in studies with large populations (45). In addition, recall bias might have affected the healthy and unhealthy items scores as they assess dietary intake during the previous day.

*Sahtak bi Sahnak* was administered and evaluated in 16 public and private secondary schools located in urban and rural regions in Lebanon, including a diverse sample. However, future studies should focus on including participants from all Lebanese regions to be able to generalise the obtained results on the whole population.

### Implications for Research, Practice, and Policy Making

*Sahtak bi Sahnak* showed substantial positive effects on the dietary behaviours and knowledge of diverse Lebanese adolescents, an understudied population. Given the great potential of the intervention, further dissemination in Lebanon and the region is recommended. The intervention can be easily integrated into the Lebanese school curriculum, and can be directly applied in schools within the context of an adolescent’s natural environment (46). This study was planned carefully to suit all Lebanese adolescents with different nutritional statuses. As 10.7% of the sample were underweight (23), the intervention focused on healthy eating habits and healthy weight concepts rather than on obesity and ideal weight. The findings may further be taken into consideration for future school-based obesity prevention programmes. Future studies should further monitor the implementation process when the intervention is disseminated to other schools, as well as the longer term effectiveness of the intervention.

## Conclusion

The current study presents the effect evaluation of a school-based intervention designed according to the IM framework, targeting Lebanese adolescents from different geographical regions. *Sahtak bi Sahnak* demonstrated its effectiveness in improving the dietary knowledge and, consequently, the dietary adherence levels of Lebanese adolescents. It also showed that this theory-based intervention is feasible to implement in public and private schools located in urban and rural areas, as the intervention succeeded in reaching our aims and in completing the intervention as planned. In addition, none of the participating schools withdrew from the programme. Future studies targeting environmental (e.g., access to healthy and unhealthy foods) and school policy changes are needed to improve eating patterns and physical activity levels at the same time.

## Data Availability Statement

The raw data supporting the conclusions of this article will be made available by the authors, without undue reservation.

## Ethics Statement

The studies involving human participants were reviewed and approved by the Lebanese Ministry of Education and Higher Education (15465/3/2016; date: 06/10/2017) and the Institutional Review Board of the Lebanese International University (LIUIRB-171212-LS1). Written informed consent from the participants’ legal guardian/next of kin was not required to participate in this study in accordance with the national legislation and the institutional requirements.

## Author Contributions

LS: conceptualisation, data collection, conducting intervention, formal analysis, investigation, and writing—original draft preparation. LS, JG, and SK: methodology, writing—review and editing, interpretation of the data, and approval of the final version. JG and SK: validation and supervision. All authors have read and agreed to the published version of the manuscript.

## Conflict of Interest

The authors declare that the research was conducted in the absence of any commercial or financial relationships that could be construed as a potential conflict of interest.

## Publisher’s Note

All claims expressed in this article are solely those of the authors and do not necessarily represent those of their affiliated organizations, or those of the publisher, the editors and the reviewers. Any product that may be evaluated in this article, or claim that may be made by its manufacturer, is not guaranteed or endorsed by the publisher.

## References

[1] World Health Organization. *Taking Action on Childhood Obesity.* Report No.: Contract No.: WHO/NMH/PND/ECHO/18.1. Geneva: World Health Organization (2018).

[2] NCD-RisC. Worldwide trends in body-mass index, underweight, overweight, and obesity from 1975 to 2016: a pooled analysis of 2416 population-based measurement studies in 128⋅9 million children, adolescents, and adults. *Lancet.* (2017) 390:2627–42. 10.1016/s0140-6736(17)32129-3 29029897 PMC5735219

[3] WHO. Prevalence of Obesity Among Children and Adolescents, BMI>+2 Standard Deviation Above the Median, crude Estimates by Country, Among Children Aged 5-19 Years. (2016). Available online at: http://apps.who.int/gho/data/node.main.BMIPLUS2C?lang=en (accessed 2020).

[4] Hruby A Hu FB. The epidemiology of obesity: a big picture. *Pharmacoeconomics.* (2015) 33:673–89. 10.1007/s40273-014-0243-x 25471927 PMC4859313

[5] Lobstein T Baur L Uauy R. Obesity in children and young people: a crisis in public health. *Obes Rev.* (2004) 5:4–85. 10.1111/j.1467-789X.2004.00133.x 15096099

[6] WHO. *Report of the Commission on Ending Childhood Obesity.* Geneva: World Health Organization (2016).

[7] Jones RA Lubans DR Morgan PJ Okely AD Parletta N Wolfenden L School-based obesity prevention interventions: practicalities and considerations. *Obes Res Clin Pract.* (2014) 8:e497–510. 10.1016/j.orcp.2013.10.004 25263839

[8] Kelsey MM Zaepfel A Bjornstad P Nadeau KJ. Age-related consequences of childhood obesity. *Gerontology.* (2014) 60:222–8. 10.1159/000356023 24434909

[9] WHO. *Report of the Commission on Ending Childhood Obesity: Implementation Plan: Executive Summary.* Report No.: Contract No.: WHO/NMH/PND/ECHO/17.1. Geneva: World Health Organization (2017).

[10] Story M Kaphingst KM French S. The role of schools in obesity prevention. *Future Child.* (2006) 16:109–42. 10.1353/foc.2006.0007 16532661

[11] Ardic A Erdogan S. The effectiveness of the COPE healthy lifestyles TEEN program: a school-based intervention in middle school adolescents with 12-month follow-up. *J Adv Nurs.* (2017) 73:1377–89. 10.1111/jan.13217 27878848

[12] Habib-Mourad C Ghandour LA Moore HJ Nabhani-Zeidan M Adetayo K Hwalla N Promoting healthy eating and physical activity among school children: findings from health-E-Pals, the first pilot intervention from Lebanon. *BMC Public Health.* (2014) 14:940. 10.1186/1471-2458-14-940 25208853 PMC4167260

[13] Ochoa-Avilés A Verstraeten R Huybregts L Andrade S Van Camp J Donoso S A school-based intervention improved dietary intake outcomes and reduced waist circumference in adolescents: a cluster randomized controlled trial. *Nutr J.* (2017) 16:79. 10.1186/s12937-017-0299-5 29228946 PMC5725778

[14] Jiang J Xia X Greiner T Wu G Lian G Rosenqvist U. The effects of a 3-year obesity intervention in schoolchildren in Beijing. *Child Care Health Dev.* (2007) 33:641–6. 10.1111/j.1365-2214.2007.00738.x 17725789

[15] Dreyhaupt J Mayer B Keis O Öchsner W Muche R. Cluster-randomized studies in educational research: principles and methodological aspects. *GMS J Med Educ.* (2017) 34:Doc26. 10.3205/zma001103 28584874 PMC5450430

[16] Chenot J-F. Cluster-randomisierte studien: eine wichtige methode in der allgemeinmedizinischen Forschung. *Z Evid Fortbild Qual Gesundhwes.* (2009) 103:475–80. 10.1016/j.zefq.2009.07.004 19839536

[17] Bartholomew-Eldredge LK. *Planning Health Promotion Programs : An Intervention Mapping Approach.* San Francisco, CA: Jossey-Bass & Pfeiffer Imprints (2016).

[18] Stok M Hoffmann S Volkert D Boeing H Ensenauer R Stelmach-Mardas M The DONE framework: creation, evaluation, and updating of an interdisciplinary, dynamic framework 2.0 of determinants of nutrition and eating. *PLoS One.* (2017) 12:e0171077. 10.1371/journal.pone.0171077 28152005 PMC5289713

[19] Said L Schneider F Kremers SPJ Gubbels JS. Application of the intervention mapping protocol to develop sahtak bi sahnak, a school-based intervention to prevent pediatric obesity among Lebanese adolescents. *Health Psychol Bull.* (2021) 5:20–38. 10.5334/hpb.27

[20] Al-Yateem N Rossiter R. Nutritional knowledge and habits of adolescents aged 9 to 13 years in Sharjah, United Arab Emirates: a crosssectional study. *East Mediterr Health J.* (2017) 23:551–8. 29105046

[21] Kresic G Kendel Jovanovic G Pavicic Zezel S Cvijanovic O Ivezic G. The effect of nutrition knowledge on dietary intake among Croatian university students. *Coll Antropol.* (2009) 33:1047–56. 20102047

[22] Wardle J Parmenter K Waller J. Nutrition knowledge and food intake. *Appetite.* (2000) 34:269–75. 10.1006/appe.1999.0311 10888290

[23] Said L Gubbels JS Kremers SPJ. Dietary knowledge, dietary adherence, and BMI of lebanese adolescents and their parents. *Nutrients.* (2020) 12:2398. 10.3390/nu12082398 32796513 PMC7468749

[24] Nasreddine L Naja F Akl C Chamieh MC Karam S Sibai A-M Dietary, lifestyle and socio-economic correlates of overweight, obesity and central adiposity in lebanese children and adolescents. *Nutrients.* (2014) 6:1038–62. 10.3390/nu6031038 24618510 PMC3967177

[25] Habib-Mourad C Ghandour LA Maliha C Awada N Dagher M Hwalla N. Impact of a one-year school-based teacher-implemented nutrition and physical activity intervention: main findings and future recommendations. *BMC Public Health.* (2020) 20:256. 10.1186/s12889-020-8351-3 32075607 PMC7031897

[26] Oldewage-Theron WH Egal A. The evaluation of a nutrition education programme on the nutrition knowledge of children aged six and seven years. *J Consum Sci.* (2009) 37:45–51.

[27] Livingston EH Cassidy L. Statistical power and estimation of the number of required subjects for a study based on the t-test: a surgeon’s primer. *J Surg Res.* (2005) 126:149–59. 10.1016/j.jss.2004.12.013 15919413

[28] Heagerty P DeLong E. *Experimental Designs and Randomization Schemes: Cluster Randomized Trials. Rethinking Clinical Trials: A Living Textbook of Pragmatic Clinical Trials.* Bethesda, MD: NIH Health Care Systems Research Collaboratory. (2020).

[29] Lee R Nieman D. *Nutritional Assessment.* 6th ed. New York, NY: McGraw-Hill Higher Education (2012).

[30] de Onis M Onyango AW Borghi E Siyam A Nishida C Siekmann J. Development of a WHO growth reference for school-aged children and adolescents. *Bull World Health Organ.* (2007) 85:660–7. 10.2471/blt.07.043497 18026621 PMC2636412

[31] Stewart L. Obesity. 4th ed. In: Shaw V editor. *Clinical Paediatric Dietetics.* Chichester: John Wiley & Sons, Ltd (2015). p. 798–808.

[32] Nihiser AJ Lee SM Wechsler H McKenna M Odom E Reinold C Body mass index measurement in schools. *J Sch Health.* (2007) 77:651–71; quiz 722–4. 10.1111/j.1746-1561.2007.00249.x 18076411

[33] Vanderwall C Randall Clark R Eickhoff J Carrel AL. BMI is a poor predictor of adiposity in young overweight and obese children. *BMC Pediatrics.* (2017) 17:135. 10.1186/s12887-017-0891-z 28577356 PMC5457636

[34] Said L Gubbels JS Kremers SPJ. Development of dietary knowledge and adherence questionnaires for lebanese adolescents and their parents. *Int J Environ Res Public Health.* (2019) 17:147. 10.3390/ijerph17010147 31878200 PMC6982025

[35] Ursachi G Horodnic IA Zait A. How reliable are measurement scales? External factors with indirect influence on reliability estimators. *Procedia Econ Finance.* (2015) 20:679–86. 10.1016/S2212-5671(15)00123-9

[36] Gidding SS Dennison BA Birch LL Daniels SR Gilman MW Lichtenstein AH Dietary Recommendations for children and adolescents a guide for practitioners: consensus statement from the American heart association. *Circulation.* (2005) 112:2061–75. 10.1161/circulationaha.105.169251 16186441

[37] Institute of Medicine. *Dietary Reference Intakes for Water, Potassium, Sodium, Chloride, and Sulfate.* Washington, DC: The National Academies Press (2005). p. 638.

[38] Becker P Carney LN Corkins MR Monczka J Smith E Smith SE Consensus statement of the academy of nutrition and dietetics/American society for parenteral and enteral nutrition: indicators recommended for the identification and documentation of pediatric malnutrition (undernutrition). *Nutr Clin Pract.* (2015) 30:147–61. 10.1177/0884533614557642 25422273

[39] Hoelscher DM Kirk S Ritchie L Cunningham-Sabo L. Position of the academy of nutrition and dietetics: interventions for the prevention and treatment of pediatric overweight and obesity. *J Acad Nutr Diet.* (2013) 113:1375–94. 10.1016/j.jand.2013.08.004 24054714

[40] Shah P Misra A Gupta N Hazra DK Gupta R Seth P Improvement in nutrition-related knowledge and behaviour of urban Asian Indian school children: findings from the ‘medical education for children/adolescents for Realistic prevention of obesity and diabetes and for healthy aGeing’ (MARG) intervention study. *Br J Nutr.* (2010) 104:427–36. 10.1017/S0007114510000681 20370939

[41] Maatoug J Msakni Z Zammit N Bhiri S Harrabi I Boughammoura L. School-based intervention as a component of a comprehensive community program for overweight and obesity prevention, Sousse, Tunisia, 2009-2014. *Prev Chronic Dis.* (2015) 12:E160. 10.5888/pcd12.140518 26402050 PMC4584471

[42] Naja F Hwalla N Fossian T Zebian D Nasreddine L. Validity and reliability of the Arabic version of the household food insecurity access scale in rural Lebanon. *Public Health Nutr.* (2015) 18:251–8. 10.1017/S1368980014000317 24702865 PMC10271416

[43] Kok G Lo SH Peters G-JY Ruiter RAC. Changing energy-related behavior: an intervention mapping approach. *Energy Policy.* (2011) 39:5280–6. 10.1016/j.enpol.2011.05.036

[44] Suresh K. An overview of randomization techniques: an unbiased assessment of outcome in clinical research. *J Hum Reprod Sci.* (2011) 4:8–11. 10.4103/0974-1208.82352 21772732 PMC3136079

[45] Kovacs E Siani A Konstabel K Hadjigeorgiou C de Bourdeaudhuij I Eiben G Adherence to the obesity-related lifestyle intervention targets in the IDEFICS study. *Int J Obes (Lond).* (2014) 38(Suppl. 2):S144–51. 10.1038/ijo.2014.145 25376216 PMC4165864

[46] Hoelscher DM Evans A Parcel G Kelder S. Designing effective nutrition interventions for adolescents. *J Am Diet Assoc.* (2002) 102(3 Suppl.):S52–63. 10.1016/S0002-8223(02)90422-0 11902389

