# Supplementary material for: Effect Evaluation of Sahtak bi Sahnak, a Lebanese Secondary School-Based Nutrition Intervention: A Cluster Randomised Trial
Source: Front Nutr. 2022 Mar 16;9:824020. doi: 10.3389/fnut.2022.824020 (PMC8966668; doi:10.3389/fnut.2022.824020)
Supplement: Supplementary file 1 [file Data_Sheet_1.pdf]

Supplementary File

Table S1. Intervention effects on the total knowledge score, healthy items score, and unhealthy items score of the indicated subgroups.

	B	CI 95%	*p*
**Total dietary knowledge score**			
Gender			
Boys	11.33	9.69-12.96	<0.001
Girls	12.76	11.74-13.77	<0.001
Location			
Urban	15.50	14.38-16.62	<0.001
Rural	10.57	9.40-11.74	<0.001
Type of school			
Public	12.79	11.75-13.82	<0.001
Private	9.34	7.68-11.00	<0.001
Grade [a]			
Grade 10	11.13	9.89-12.36	<0.001
Grade 11	13.65	12.52-14.78	<0.001
Nutritional status [a]			
Malnourished	15.36	12.82-17.89	<0.001
Healthy weight	12.52	11.41-13.62	<0.001
Overweight/ obese	10.61	9.03-12.19	<0.001
Dietary knowledge level at baseline [b]			
Low	13.48	12.26-14.70	<0.001
Acceptable	9.87	8.42-11.32	<0.001
**Healthy items score**			
Gender			
Boys	1.28	0.64-1.93	<0.001
Girls	2.13	1.61-2.64	<0.001
Location [a]			
Urban	3.26	2.68-3.85	<0.001
Rural	0.77	0.26-1.29	0.003
Type of school			
Public	1.64	1.17-2.11	<0.001
Private	2.13	1.35-2.90	<0.001
Grade			
Grade 10	1.44	0.88-1.99	<0.001
Grade 11	1.97	1.43-2.52	<0.001
Nutritional status			
Underweight	1.29	0.20-2.38	0.021
Healthy weight	1.75	1.24-2.27	<0.001
Overweight/ obese	1.92	1.19-2.65	<0.001

Table S1. (continued)

	B	CI 95%	*p*
Unhealthy items score			
Gender			
Boys	-1.92	(-2.46) - (-1.38)	<0.001
Girls	-1.14	(-1.54) - (-0.73)	<0.001
Location			
Urban	-1.53	(-2.03) - (-1.03)	<0.001
Rural	-1.30	(-1.70) - (-0.89)	<0.001
Type of school			
Public	-1.60	(-1.98) - (-1.21)	<0.001
Private	-0.85	(-1.48) - (-0.23)	0.008
Grade			
Grade 10	-1.51	(-1.96) - (-1.07)	<0.001
Grade 11	-1.38	(-1.83) - (-0.93)	<0.001
Nutritional status			
Underweight	-0.88	(-1.84) - (0.08)	0.073
Healthy weight	-1.26	(-1.69) - (-0.84)	<0.001
Overweight/ obese	-1.86	(-2.41) - (-1.31)	<0.001

Note: Multivariate regression model was performed to test the intervention effect on the total knowledge score, healthy items score, and unhealthy items score in the indicated subgroups. All models were adjusted for age, gender, class, type of school (public vs. private), location (urban vs. rural), BMI *z*-score, and score at baseline. BMI classification: adolescents with a BMI *z*-score ≤ -1 were classified as underweight; adolescents with -0.99 ≤ BMI z-score ≤ 1.03 were classified as having a healthy weight; and adolescents with a BMI z-score ≥ 1.04 were considered overweight/obese. The interaction between background variables and the intervention variable was found to be significantly associated with the dependent variables (i.e., knowledge score and healthy items score) with [a] $p<0.05$ and [b] $p<0.001$. Abbreviations: CI: Confidence interval.

Table S2. Intervention effects of the separate items of the Dietary Adherence Questionnaire.

DAQ items	B	CI 95%	*p*
Lean meat	0.16	0.08-0.24	<0.001
High fat meat	-0.25	-0.33-0.17	<0.001
Unhealthy snacks	-0.15	-0.23-(-0.07)	<0.001
Low-fat dairy products	0.06	-0.02-0.14	0.119
Medium fat dairy products	0.02	-0.04-0.07	0.573
High-fat dairy products	-0.07	-0.16-0.01	0.086
Whole milk	0.09	0.03-0.15	0.004
Skimmed milk	-0.005	-0.01-0.003	0.264
Refined bread	-0.13	-0.25-(-0.01)	0.027
Whole bread	0.04	-0.01-0.09	0.089
French fries	-0.13	-0.19-(-0.06)	<0.001
Beans	0.04	-0.02-0.10	0.163
Vegetables	0.11	0.02-0.20	0.016
Fruits	-0.001	-0.08-0.08	0.985
Natural juices	-0.001	-0.04-0.04	0.957
Commercial juices	-0.14	-0.19-(-0.08)	<0.001
Soft drinks	-0.20	-0.28-(-0.12)	<0.001
Diet soft drinks	-0.01	-0.02-0	0.059
Energy drinks	-0.01	-0.04-0.01	0.419
Sweets	-0.18	-0.26-(-0.09)	<0.001
Arabic sweets	0.01	-0.01-0.04	0.210
Breakfast	0.29	0.23-0.35	<0.001
Number of meals	0.24	0.14-0.33	<0.001
Number of snacks	-0.29	-0.41-(-0.18)	<0.001
Number of meals outside	-0.09	-0.14-(-0.04)	<0.001
PA at school	-0.10	-0.06-0.02	0.359
PA outside of the school	-0.02	0.15-0.26	<0.001
PA weekly hours	0.26	0.17-0.36	<0.001
Screen-viewing duration	-0.47	-0.68-(-0.25)	<0.001
Smoking	-0.03	-0.07-0.01	0.129

Multivariate regression model was performed to test the intervention effect on the separate items of the dietary adherence questionnaire. All models were adjusted for age, gender, class, type of school (public vs. private), location (urban vs. rural), BMI *z*-score, and score at baseline. Abbreviations: CI: Confidence interval, DAQ: Dietary adherence questionnaire, PA: Physical activity

Table S3: CONSORT 2010 checklist of information to include when reporting a cluster randomised trial

Section/Topic	Item No	Standard Checklist item	Extension for cluster designs	Page No *
**Title and abstract**				
	1a	Identification as a randomised trial in the title	Identification as a cluster randomised trial in the title	1
	1b	Structured summary of trial design, methods, results, and conclusions (for specific guidance see CONSORT for abstracts)[i,ii]	See table 2	1
**Introduction**				
**Background and objectives**	2a	Scientific background and explanation of rationale	Rationale for using a cluster design	2
	2b	Specific objectives or hypotheses	Whether objectives pertain to the cluster level, the individual participant level or both	2
**Methods**				
**Trial design**	3a	Description of trial design (such as parallel, factorial) including allocation ratio	Definition of cluster and description of how the design features apply to the clusters	2-4
	3b	Important changes to methods after trial commencement (such as eligibility criteria), with reasons		Not applicable
**Participants**	4a	Eligibility criteria for participants	Eligibility criteria for clusters	3
	4b	Settings and locations where the data were collected		**3**
**Interventions**	5	The interventions for each group with sufficient details to allow replication, including how and when they were actually administered	Whether interventions pertain to the cluster level, the individual participant level or both	4-5
**Outcomes**	6a	Completely defined pre-specified primary and secondary outcome measures, including how and when they were assessed	Whether outcome measures pertain to the cluster level, the individual participant level or both	4
	6b	Any changes to trial outcomes after the trial commenced, with reasons		Not applicable

Table S3. (continued)

Section/Topic	Item No	Standard Checklist item	Extension for cluster designs	Page No *
**Sample size**	7a	How sample size was determined	Method of calculation, number of clusters(s) (and whether equal or unequal cluster sizes are assumed), cluster size, a coefficient of intracluster correlation (ICC or *k*), and an indication of its uncertainty	2-3
	7b	When applicable, explanation of any interim analyses and stopping guidelines		Not applicable
**Randomisation:**				
**Sequence generation**	8a	Method used to generate the random allocation sequence		**3-4**
	8b	Type of randomisation; details of any restriction (such as blocking and block size)	Details of stratification or matching if used	3-4
**Allocation concealment mechanism**	9	Mechanism used to implement the random allocation sequence (such as sequentially numbered containers), describing any steps taken to conceal the sequence until interventions were assigned	Specification that allocation was based on clusters rather than individuals and whether allocation concealment (if any) was at the cluster level, the individual participant level or both	4
**Implementation**	10	Who generated the random allocation sequence, who enrolled participants, and who assigned participants to interventions	Replace by 10a, 10b and 10c	3-4
	10a		Who generated the random allocation sequence, who enrolled clusters, and who assigned clusters to interventions	
	10b		Mechanism by which individual participants were included in clusters for the purposes of the trial (such as complete enumeration, random sampling)	
	10c		From whom consent was sought (representatives of the cluster, or individual cluster members, or both), and whether consent was sought before or after randomisation	

Table S3. (continued)

Section/Topic	Item No	Standard Checklist item	Extension for cluster designs	Page No *
**Blinding**	11a	If done, who was blinded after assignment to interventions (for example, participants, care providers, those assessing outcomes) and how		Not applicable
	11b	If relevant, description of the similarity of interventions		Not applicable
**Statistical methods**	12a	Statistical methods used to compare groups for primary and secondary outcomes	How clustering was taken into account	5
	12b	Methods for additional analyses, such as subgroup analyses and adjusted analyses		5
**Results**				
**Participant flow (a diagram is strongly recommended)**	13a	For each group, the numbers of participants who were randomly assigned, received intended treatment, and were analysed for the primary outcome	For each group, the numbers of clusters that were randomly assigned, received intended treatment, and were analysed for the primary outcome	Figure
	13b	For each group, losses and exclusions after randomisation, together with reasons	For each group, losses and exclusions for both clusters and individual cluster members	Figure
**Recruitment**	14a	Dates defining the periods of recruitment and follow-up		5
	14b	Why the trial ended or was stopped		Not applicable
**Baseline data**	15	A table showing baseline demographic and clinical characteristics for each group	Baseline characteristics for the individual and cluster levels as applicable for each group	Table 1
**Numbers analysed**	16	For each group, number of participants (denominator) included in each analysis and whether the analysis was by original assigned groups	For each group, number of clusters included in each analysis	5

Table S3. (continued)

Section/Topic	Item No	Standard Checklist item	Extension for cluster designs	Page No *
**Outcomes and estimation**	17a	For each primary and secondary outcome, results for each group, and the estimated effect size and its precision (such as 95% confidence interval)	Results at the individual or cluster level as applicable and a coefficient of intracluster correlation (ICC or k) for each primary outcome	6<br>Table 2
	17b	For binary outcomes, presentation of both absolute and relative effect sizes is recommended		
**Ancillary analyses**	18	Results of any other analyses performed, including subgroup analyses and adjusted analyses, distinguishing pre-specified from exploratory		6<br>Table 3
**Harms**	19	All important harms or unintended effects in each group (for specific guidance see CONSORT for harms[iii])		Not applicable
**Discussion**				
**Limitations**	20	Trial limitations, addressing sources of potential bias, imprecision, and, if relevant, multiplicity of analyses		7
**Generalisability**	21	Generalisability (external validity, applicability) of the trial findings	Generalisability to clusters and/or individual participants (as relevant)	7
**Interpretation**	22	Interpretation consistent with results, balancing benefits and harms, and considering other relevant evidence		7-8

Table S3. (continued)

Section/Topic	Item No	Standard Checklist item	Extension for cluster designs	Page No *
**Other information**				
**Registration**	23	Registration number and name of trial registry		Not applicable Study protocol approved by the Lebanese Ministry of Education and Higher Education (15465/3/2016; date: 06/10/2017) and the Institutional Review Board of the Lebanese International University (LIUIRB-171212-LS1).
**Protocol**	24	Where the full trial protocol can be accessed, if available		Said L, Schneider F, Kremers SPJ, Gubbels JS. Application of the Intervention Mapping Protocol to Develop Sahtak bi Sahnak, a School-Based Intervention to Prevent Pediatric Obesity among Lebanese Adolescents. Health Psychology Bulletin (2021) 5(1):20–38. doi: http://doi.org/10.5334/hpb.27
**Funding**	25	Sources of funding and other support (such as supply of drugs), role of funders		8

** Note: page numbers optional depending on journal requirements*
Checklist downloaded from: www.consort-statement.org

i Hopewell S, Clarke M, Moher D, Wager E, Middleton P, Altman DG, et al. CONSORT for reporting randomised trials in journal and conference abstracts. *Lancet* 2008, 371:281-283

ii Hopewell S, Clarke M, Moher D, Wager E, Middleton P, Altman DG at al (2008) CONSORT for reporting randomized controlled trials in journal and conference abstracts: explanation and elaboration. *PLoS Med* 5(1): e20

iii Ioannidis JP, Evans SJ, Gotzsche PC, O'Neill RT, Altman DG, Schulz K, Moher D. Better reporting of harms in randomized trials: an extension of the CONSORT statement. *Ann Intern Med* 2004; 141(10):781-788.
